# Memantine Confers Multi‐Target Protection in a Zebrafish Seizure Model: Attenuating Epileptic Behavior, GluN2A Overexpression, and Oxidative Stress

**DOI:** 10.1111/jnc.70345

**Published:** 2026-01-02

**Authors:** Kamila Cagliari Zenki, Eduardo Kalinine, Ben Hur Marins Mussulini, Thainá Garbino dos Santos, Lucia von Mengden, Fábio Klamt, Suelen Baggio, Ana Carolina de Moura, Ana Beatriz Gorini da Veiga, Diogo Losch de Oliveira

**Affiliations:** ^1^ Laboratory of Neural Development, Department of Biochemistry, Instituto de Ciências Básicas da Saúde Universidade Federal do Rio Grande do Sul Porto Alegre Brazil; ^2^ Programa de Pós‐graduação em Ciências Biológicas: Bioquímica, Instituto de Ciências Básicas da Saúde Universidade Federal do Rio Grande do Sul Porto Alegre Brazil; ^3^ Centre of New Technologies University of Warsaw Warszawa Poland; ^4^ Laboratory of Cellular Biochemistry, Department of Biochemistry, Instituto de Ciências Básicas da Saúde Universidade Federal do Rio Grande do Sul Porto Alegre Brazil; ^5^ Laboratory of Molecular Biology Universidade Federal de Ciências da Saúde de Porto Alegre—UFCSPA Porto Alegre Brazil

**Keywords:** anxiety, drug repurposing, memantine, seizure, zebrafish

## Abstract

Drug repurposing represents a strategic approach to identifying multi‐target therapies for complex disorders like refractory epilepsy. Memantine (MN), a well‐tolerated N‐methyl‐D‐aspartate receptor (NMDAR) antagonist with additional multi‐target activities, is a promising candidate for repurposing. This study investigated the preventive effects of MN on pentylenetetrazol (PTZ)‐induced seizures and its associated neurochemical and behavioral sequelae in adult zebrafish. Animals were pre‐treated with MN (20 or 50 mg/kg, i.p.) or vehicle 1 or 2 h before PTZ exposure. Seizure behavior was assessed immediately, while neurochemical and behavioral analyses were conducted 24 h post‐seizure. MN pre‐treatment significantly attenuated seizure severity and delayed the onset of tonic–clonic seizures. Notably, MN prevented the PTZ‐induced upregulation of the GluN2A NMDAR subunit and mitigated oxidative stress by reducing protein carbonylation and normalizing superoxide dismutase (SOD) activity. Furthermore, MN abolished the PTZ‐induced increase in time spent in the white compartment of a light/dark test, a behavioral indicator of disrupted defensive responses. These results demonstrate that MN confers robust anticonvulsant, neuroprotective, and behavioral‐stabilizing effects in a zebrafish seizure model. Our findings reinforce the potential of memantine as a novel multi‐target adjunct therapy for mitigating the neurobehavioral consequences of epilepsy.

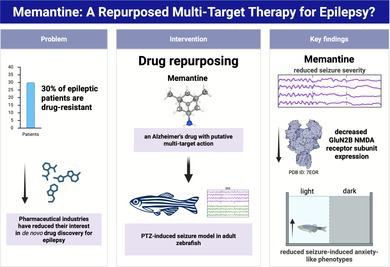

## Graphical abstract

Approximately 30% of epilepsy patients do not respond to current medications. In addition, the pharmaceutical industry is reducing its interest in de novo drug discovery for epilepsy. Drug repurposing offers a promising alternative. This study investigated memantine, an Alzheimer's drug, in a zebrafish model of seizures. We found that memantine not only reduced seizure severity but also prevented key molecular consequences: the overproduction of a specific brain receptor (the GluN2A NMDA receptor subunit) and oxidative brain damage. Importantly, it also alleviated seizure‐induced anxiety‐like behavior. Our findings position memantine as a compelling multi‐target candidate for repurposing to treat epilepsy and its debilitating neuropsychiatric co‐morbidities.

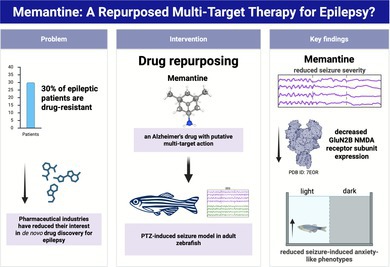

**Abbreviations**
AEDs: antiepileptic drugs
DNPH: 2,4‐dinitrophenylhydrazine
DTNB: 5,5′‐dithiobis‐(2‐nitrobenzoic acid)
GluN1: N‐methyl‐D‐aspartate receptor subunit 1
GluN2A: N‐methyl‐D‐aspartate receptor subunit 2A
GluN2B: N‐methyl‐D‐aspartate receptor subunit 2B
GPx: glutathione peroxidase
GSH: reduced glutathione
MN: memantine
NMDAR: N‐methyl‐D‐aspartate receptor
OPA: o‐phthaldialdehyde
PTZ: pentylenetetrazol
RRID: Research Resource Identifier
SOD: superoxide dismutase

## 1 Introduction

Generalized seizures can induce long‐term and irreversible neurological consequences (Trinka et al. 2015). While current antiepileptic drugs (AEDs) are clinically effective at terminating convulsive episodes (Glauser et al. 2016), they often fail to prevent the underlying neurochemical alterations associated with seizure‐induced brain damage and the development of epilepsy. Furthermore, these therapies are frequently associated with undesirable side effects (Kalemenev et al. 2016; Trinka et al. 2015). This therapeutic inadequacy is underscored by the fact that approximately 30% of patients develop drug‐resistant epilepsy, which remains a significant clinical and economic burden (Brodie et al. 2012). With declining industrial interest in de novo drug discovery for epilepsy, a promising strategy is to investigate approved drugs for new therapeutic applications, particularly focusing on multi‐target agents (Löscher et al. 2013).

Drug repurposing, the identification of new therapeutic uses for existing drugs, offers a strategic alternative. This approach presents a higher probability of success at preclinical and clinical stages compared to developing new chemical entities, while also reducing associated costs and timelines (Barratt 2012; Ashburn and Thor 2004). It has fostered collaborative platforms and partnerships between academia and industry to identify promising candidate molecules (Caban et al. 2017; Murteira et al. 2014). Repurposing is especially valuable for central nervous system disorders, where multi‐target therapies are increasingly recognized as a viable solution for complex conditions like refractory epilepsy (Morphy et al. 2004; Rocha et al. 2023).

In this context, memantine (MN) emerges as a prominent example of a repurposed, multi‐target ligand for CNS disorders (Lipton 2006; Zheng et al. 2014). An FDA‐approved drug for moderate‐to‐severe Alzheimer's disease (Schuh and Khanna 2023), MN is a well‐tolerated, uncompetitive antagonist of the N‐methyl‐D‐aspartate receptor (NMDAR). Its favorable clinical profile is attributed to its fast blocking/unblocking kinetics and low‐to‐moderate affinity, which permit a partial blockade that mitigates excitotoxicity without completely disrupting the physiological synaptic functions of NMDARs (Glasgow et al. 2017; Parsons et al. 2008). Beyond NMDAR antagonism, MN exhibits uncompetitive antagonism at serotonin 5‐HT3 receptors (Rammes et al. 2001) and acts as an agonist at dopamine D2 receptors (Seeman et al. 2008), with affinities comparable to its action on NMDARs. Recent evidence further suggests that MN modulates redox imbalance in various pathological states, bolstering its profile as a multi‐target agent (Bardak et al. 2018; Khalili‐Fomeshi et al. 2018; Tanaka et al. 2018).

Emerging preclinical study developed by our research group supports the anticonvulsant potential of MN. For instance, administration of MN (20 mg/kg) was shown to be neuroprotective, attenuate seizure severity, and confer a unique profile of neuroprotection across the immature brain, even when administered post‐ictally in a lithium‐pilocarpine model of status epilepticus in rats (Zenki et al. 2018).

The zebrafish ( *Danio rerio* ) has emerged as a powerful model organism for translational epilepsy research (Baraban 2007; Berghmans et al. 2007; Mussulini et al. 2016). Its considerable genetic homology to humans (approximately 84% of genes linked to human disease have a zebrafish counterpart) (Howe et al. 2013), conserved CNS architecture (Kalueff et al. 2014), and high degree of physiological relevance provide a strong foundation for modeling neurological disorders (Kalueff et al. 2014). The species offers significant experimental advantages, including external development for facile genetic manipulation (Koster and Sassen 2015), permeability to water‐soluble compounds for drug screening, and well‐characterized behavioral phenotypes (Kalueff 2017). Critically, mutations in orthologs of human epileptic genes can induce spontaneous seizures, faithfully recapitulating convulsive phenotypes (Griffin et al. 2016). These attributes, combined with compatibility with medium‐ to high‐throughput formats, establish zebrafish as a unique and indispensable system for investigating epileptogenic mechanisms and for the early‐stage discovery and validation of novel anti‐seizure therapies.

Given that seizure activity is strongly linked to NMDAR overactivation and subsequent neurochemical dysregulation, and considering MN's favorable safety profile and multi‐target mechanism of action, we hypothesized that MN would confer protection against pentylenetetrazol (PTZ)‐induced seizures in adult zebrafish. Therefore, the aim of the present study was to investigate the potential of MN to prevent PTZ‐induced seizures in adult zebrafish and to evaluate its ability to mitigate the associated neurochemical and behavioral deficits.

## 2 Methods

### 2.1 Drugs

Pentylenetetrazole (cat#P6500), tricaine (cat#A5040), and memantine hydrochloride (cat#M9292) were purchased from Sigma‐Aldrich (USA). Trizol reagent (cat#15596026), M‐MLV Reverse Transcriptase (200 U/μL) (cat#28025013), and SYBR Green chemistry (cat#43‐444‐63) were purchased from Invitrogen Life Technologies (USA). Other chemicals were purchased from a local supplier.

### 2.2 Animal Husbandry and Ethical Approval

A total of 488 adult wild‐type zebrafish ( *Danio rerio* ) (short‐fin strain; 4–6 months old; sex ratio was close to 50:50 male/female; RRID:ZIRC_ZL1) were obtained from a commercial supplier (Delphis, Porto Alegre, Brazil). Following a minimum 2‐week acclimation period, fish were housed in 8 L tanks at an automated recirculating system (Zebtec, Tecniplast, Italy). The housing system was equipped with mechanical, chemical and biological filtration. Water parameters were maintained as follows: temperature 28°C ± 1°C, pH 7.0 ± 1, and conductivity 500 ± 100 μS/cm. A 14/10 h light/dark cycle (lights on at 7:00 am) was provided by ceiling‐mounted fluorescent lamps, delivering an intensity of 600 lx at the water's surface. Fish were fed four times daily with a combination of ZebraFeed flake food (Sparos, Portugal) and freshly hatched *Artemia* sp. Nauplii (two times a day each). Only fishes with a body weight of 0.25–0.35 g were used in experiments.

All experimental procedures were conducted in accordance with the Brazilian Law for Care and Use of Laboratory Animals (Law 11794/2008) and were previously approved by the Committee for Animal Care and Use of Universidade Federal do Rio Grande do Sul (number 34097).

### 2.3 Drug Administration and Experimental Design

Following anesthesia by immersion in a 160 μg/mL tricaine solution (until complete immobility and reduced opercular movement were observed; 1–2 min) (Alfaro et al. 2011), fish received an intraperitoneal (i.p.) injection of either memantine (MN; 20 or 50 mg/kg) or vehicle (0.9% saline). The 20 mg/kg dose of memantine was selected based on its FDA‐approved maximum daily dose for Alzheimer's disease (Kennedy et al. 2018; Rogawski and Wenk 2003). A higher, non‐clinical dose of 50 mg/kg was based on previous reports suggesting potential neurobiological benefits at this dosage in preclinical rodent studies, such as enhancing cell proliferation (Zisiadis et al. 2023), elevating BDNF mRNA expression in the limbic cortex (Marvanová et al. 2001), and reducing self‐administration in ethanol‐dependent rats (Alaux‐Cantin et al. 2015). The maximum injection volume did not exceed 10 μL/g of body mass (Kinkel et al. 2010). The experiments were not conducted with blinding.

An initial experiment was conducted to assess the sedative or hypolocomotor effects of MN. Animals (*N* = 10 animals/group; total of 120 animals) were treated with vehicle (NaCl 0.9% solution), MN 20 mg/kg, or MN 50 mg/kg, and their locomotor activity was monitored in the novel tank test (Figure S1A). As MN significantly reduced the distance traveled at the 0‐ and 30‐min time points post‐injection (Figure S1B), subsequent anticonvulsant testing was performed at 1 and 2 h after MN administration to allow for the dissipation of acute sedative effects.

To evaluate the anticonvulsant effects of MN, a separate cohort of animals (*N* = 92 animals/group; total of 368 animals) was pre‐treated with vehicle or MN (20 or 50 mg/kg; i.p.) 1 or 2 h prior to immersion in system water (control) or system water + 10 mM PTZ solution (experimental design was depicted in Figure S2). Seizure behavior was assessed over a 20‐min observation period according to a previously established protocol of our research group (Mussulini et al. 2013). Following the test, fish were returned to their home tanks.

Twenty‐four hours post‐PTZ exposure, animals from each group were divided into two subsets for neurochemical (qRT‐PCR and oxidative stress damage, *N* = 36 animals/treatment group) and behavioral analyses (anxiety‐like behavior, *N* = 10 animals/treatment group). For neurochemical assessments, animals were anesthetized by immersion in a 160 μg/mL tricaine solution at 4°C until complete immobility and cessation of opercular movement. Their whole brains were rapidly dissected, flash‐frozen in liquid nitrogen, and stored at −80°C until processing. All experiments were performed in three independent replicates.

### 2.4 RNA Extraction, cDNA Synthesis, and Quantitative Real‐Time PCR (qRT‐PCR)

#### 2.4.1 RNA Extraction and Quantification

Total RNA was isolated from frozen brain tissue (*N* = 6 samples/group; each sample comprising a pool of two zebrafish brains) using Trizol reagent (Invitrogen Life Technologies, USA) according to the manufacturer's instructions. Briefly, samples were homogenized in Trizol, mixed with chloroform (1:5, v/v), and centrifuged at 12 000× *g* for 15 min at 4°C. The aqueous phase was collected, and RNA was precipitated with an equal volume of isopropanol for 10 min at room temperature, followed by centrifugation (12 000× *g*, 10 min, 4°C). The resulting pellet was washed twice with 75% ethanol, centrifuged (7500× *g*, 5 min, 4°C), and air‐dried. The purified RNA was dissolved in DEPC‐treated water, quantified spectrophotometrically (NanoDrop ND‐1000; RRID:SCR_016517) to assess purity (A260/A280 and A260/A230 ratios), and stored at −80°C.

#### 2.4.2 cDNA Synthesis

One microgram of total RNA from each sample was used for cDNA synthesis. RNA was first incubated with 1 μL of oligo (dT) primer (0.5 μg/μL, Invitrogen) and 1 μL of dNTP mix (10 mM) at 65°C for 5 min in a final volume of 13 μL and then chilled on ice. A master mix containing 4 μL of 5× RT buffer (50 mM Tris–HCl, pH 8.3, 75 mM KCl, 3 mM MgCl_2_), 2 μL of 0.1 M DTT, and 1 μL of M‐MLV Reverse Transcriptase (200 U/μL) was added to a final reaction volume of 20 μL. The synthesis was performed at 37°C for 50 min, followed by enzyme inactivation at 70°C for 15 min.

#### 2.4.3 Quantitative PCR (qPCR)

The relative mRNA expression of NMDA receptor subunits (*grin1a* [GluN1], *grin2a* [GluN2A], and *grin2b* [GluN2B]) was quantified by qPCR using SYBR Green chemistry (Applied Biosystems) on a StepOnePlus Real‐Time PCR System (Applied Biosystems, USA) (RRID:SCR_015805). *β‐actin* was used as the reference gene due to its established expression stability in the zebrafish brain (Casadei et al. 2011).

Gene‐specific primers (Table 1) were designed using Primer3Plus software based on zebrafish sequences from the GenBank database and previously published work (Hunt et al. 2012). Primer specificity was confirmed by BLAST analysis (NCBI) and by the presence of a single peak in melting curve analysis. A standard curve from serial cDNA dilutions (1:1, 1:5, 1:25, 1:125, 1:625, 1:3125) was generated for each primer pair to calculate amplification efficiency (E = 10^(−1/slope) − 1); only primers with efficiencies between 90% and 110% were used (Svec et al. 2015).

**TABLE 1:** Primers sequences used for qPCR assays.

Gene name	Gene symbol	Primer sequence (5′–3′)
β‐actin	β‐actin	GCATTGCTGACCGTATGCAG (F)
		GCCAGACTCATCGTACTCCTG (R)
Glutamate ionotropic receptor NMDA type subunit 1a	Grin1a	CGAGCCCAAGATTGTGAACA (F)
		CCTGGGTGACGGCATCTTTA (R)
Glutamate ionotropic receptor NMDA type subunit 2a	Grin2a	AAAAACTACCCAGCCATGCAC (F)
		CAATGCCGTATCCTGTTGTG (R)
Glutamate ionotropic receptor NMDA type subunit 2b	Grin2b	CGTGGAGGACGTGGACCCGC (F)
		GACGGCCGAGTCCTGCGTCTG (R)

Abbreviations: F, primer forward; R, primer reverse.

Each 15 μL qPCR reaction contained 7.5 μL of SYBR Green PCR Master Mix, 0.5 μL of each forward and reverse primer (10 μM), 1 μL of cDNA template, and 5.5 μL of nuclease‐free water. The thermal cycling protocol consisted of an initial denaturation at 95°C for 10 min, followed by 40 cycles of 95°C for 30 s, 60°C for 40 s, and 72°C for 40 s. All reactions were performed in duplicate, and no‐template controls were included in each run.

Cycle threshold (CT) values were determined using the automated threshold setting in the StepOnePlus software (v2.3). Gene expression was calculated using the 2^(–ΔΔCT) method (Livak and Schmittgen 2001), with the control group (vehicle‐treated) used as the calibrator.

### 2.5 Oxidative Stress and Antioxidant Defense Assays

Frozen brain tissue samples (*N* = 4 samples/group; each sample comprising a pool of six zebrafish brains) were homogenized in 1000 μL of lysis buffer (50 mM Tris–HCl, pH 8.0, 0.5% Igepal CA‐630) and centrifuged at 600× *g* for 2 min at 4°C. The supernatant was collected, and the total protein concentration was determined as follows (Section 2.6). All samples were normalized to a uniform concentration of 1 μg/μL for subsequent analyses.

#### 2.5.1 Total Reduced Thiol (‐SH) Assay

Total reduced thiol (‐SH) content was quantified using Ellman's reagent (5,5′‐dithiobis‐(2‐nitrobenzoic acid); DTNB) (Ellman 1959). Briefly, duplicate aliquots of each sample (50 μL) were reacted with DTNB, and the absorbance was measured at 412 nm. Thiol concentration was calculated using a molar extinction coefficient of 14 150 M^−1^ cm^−1^ and expressed as μmol of ‐SH per mg of protein.

#### 2.5.2 Protein Carbonyl Content Assay

Protein carbonylation, a marker of oxidative protein damage, was assessed using the 2,4‐dinitrophenylhydrazine (DNPH) method with modifications (Yan et al. 1995). Sample aliquots (200 μL) were derivatized with 10 mM DNPH for 1 h at room temperature. Proteins were then precipitated and washed with a series of ethanol and ethyl acetate washes to remove free DNPH. The final pellet was dissolved in 3% SDS, and the absorbance of the hydrazone derivative was measured at 370 nm. Results were calculated using a molar extinction coefficient of 22 000 M^−1^ cm^−1^ and expressed as nmol of carbonyl groups per mg of protein.

#### 2.5.3 Superoxide Dismutase (SOD) Activity

SOD activity was measured based on its ability to inhibit the autoxidation of epinephrine to adrenochrome at alkaline pH (Misra and Fridovich 1972). Samples (10, 20, and 40 μL) were added to 50 mM glycine‐NaOH buffer (pH 10). The reaction was initiated by adding epinephrine, and the increase in absorbance was monitored kinetically at 480 nm for 20 min at 32°C. One unit of SOD activity is defined as the amount of enzyme that inhibits the rate of epinephrine autoxidation by 50%. Results are expressed as SOD units per mg of protein.

#### 2.5.4 Catalase Activity

Catalase activity was determined by monitoring the decomposition of hydrogen peroxide (H_2_O_2_) at 240 nm. Duplicate sample aliquots (10 μL) were added to 50 mM phosphate buffer (pH 7.0). The reaction was initiated by adding H_2_O_2_ (final concentration 10–50 mM), and the decrease in absorbance was recorded for 1 min at 25°C. Activity was calculated using the molar extinction coefficient for H_2_O_2_ (43.6 M^−1^ cm^−1^). One unit of catalase is defined as the amount that decomposes 1 μmol of H_2_O_2_ per minute per mg of protein.

#### 2.5.5 Glutathione Peroxidase (GPx) Activity

GPx activity was assayed by a coupled enzymatic method. The assay measures the oxidation of NADPH at 340 nm in a reaction mixture containing reduced glutathione (GSH), glutathione reductase, sodium azide (to inhibit catalase), and tert‐butyl hydroperoxide as a substrate. Sample aliquots (30 μL) were added to the reaction mixture, and the linear decrease in absorbance was recorded. Activity was calculated using the molar extinction coefficient of NADPH (6220 M^−1^ cm^−1^). One unit of GPx activity is defined as the amount of enzyme that oxidizes 1 μmol of NADPH per minute per mg of protein.

#### 2.5.6 GSH Concentration Assay

The concentration of GSH was quantified fluorometrically using o‐phthaldialdehyde (OPA) as described by Browne and Armstrong (1998) with modifications. Sample aliquots (30 μL) were deproteinized with metaphosphoric acid and centrifuged. The resulting supernatant was reacted with OPA (1 mg/mL) in 100 mM sodium phosphate buffer (pH 8.0, containing 5 mM EDTA) for 15 min in the dark. Fluorescence was measured at an excitation/emission of 350/420 nm. GSH concentration was determined from a standard curve (0.001–1 mM) and expressed as nmol GSH per mg of protein.

### 2.6 Protein Quantification

Total protein content was quantified using a modified Lowry assay (Peterson 1977).

### 2.7 Behavioral Analysis

Behavioral evaluations following PTZ‐induced seizures were conducted between 9:00 and 12:00 a.m. To minimize external stress and potential experimental bias, environmental interferents such as noise, vibration, and movement near the tanks were strictly controlled. Fish were acclimated to the experimental room for 30 min prior to testing. Subsequently, animals were gently netted and transferred to the behavioral apparatus. The behavior of each fish was recorded and analyzed using the ANY‐maze video‐tracking system (Stoelting Co., USA).

#### 2.7.1 Light/Dark Test

Animals (*N* = 10 animals/group) were individually tested in a light/dark box assay, adapted from Córdova et al. (2016). The apparatus consisted of a rectangular tank (30 L × 15 D × 10 H cm) divided equally into two compartments: one white and illuminated (light zone) and one black (dark zone). Light intensity in the center of each compartment was set up to 70 lx. The walls and floor of both compartments were covered with opaque, self‐adhesive plastic film. The tank was filled with system water, and the water column depth was 4 cm. Each fish was introduced into the light zone and allowed to explore the apparatus freely for 15 min. The time spent in each compartment and the number of transitions between them were recorded.

### 2.8 Statistical Analysis

All data were tested for normality by D'Agostino‐Pearson omnibus normality test. Normal distributed data were expressed as mean with 95% confidence interval (CI) and were analyzed by two‐way ANOVA followed by Tukey's post hoc test. Seizure scores presented in Figure S3 were presented as median with interquartile range. *p* ≤ 0.05 was considered significantly. All statistical data were reported in the Table S1 (Supporting Information file).

The sample size was calculated using the “pwrss” package (v1.0.0) in the R language, with the following criteria: number of factor A = 3, number of factor B = 2, *α* = 0.05, test power = 0.8, effect size = 0.5. The total sample size was 368 fish. These animals were randomly assigned to experimental groups using a computer‐generated sequence created with the “randomizr” (v1.0.0) and “dplyr” (v1.1.4) packages in R. The complete script for both sample size calculation and randomization is provided in the Supporting Information file.

## 3 Results

### 3.1 Memantine Attenuates PTZ‐Induced Seizure Severity

**FIGURE 1:**
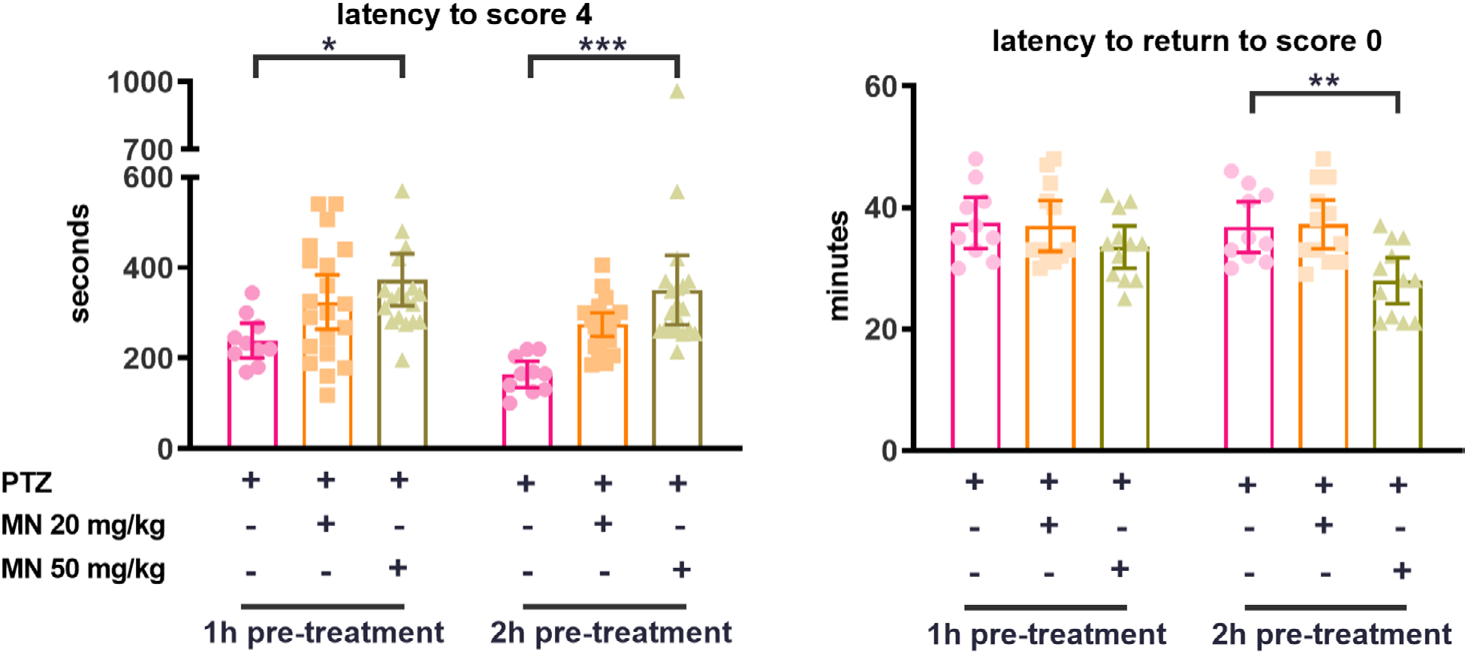
Preventive anticonvulsant action of memantine on PTZ‐induced seizures. Data were expressed as mean with 95% confidence interval (CI) and analyzed by two‐way ANOVA followed by Tukey's *post hoc* test. *p < 0.05; **p < 0.01, and ***p < 0.001.

The latency to stage 4 (tonic–clonic seizures) in vehicle/PTZ‐treated animals was consistent with previously reported values (Mussulini et al. 2013) (Figure 1). Pre‐treatment with memantine at 50 mg/kg significantly increased this latency at both pre‐treatment time points (*F*(2, 94) = 13.46, *p* < 0.0001). Furthermore, animals pre‐treated with memantine (50 mg/kg, 2 h) exhibited a significantly shorter recovery time (latency to return to stage 0) compared to the vehicle/PTZ group (*F*(2, 62) = 8.667, *p* = 0.0005). The lower dose of MN (20 mg/kg) did not produce significant effects on these evaluations.

**FIGURE 2:**
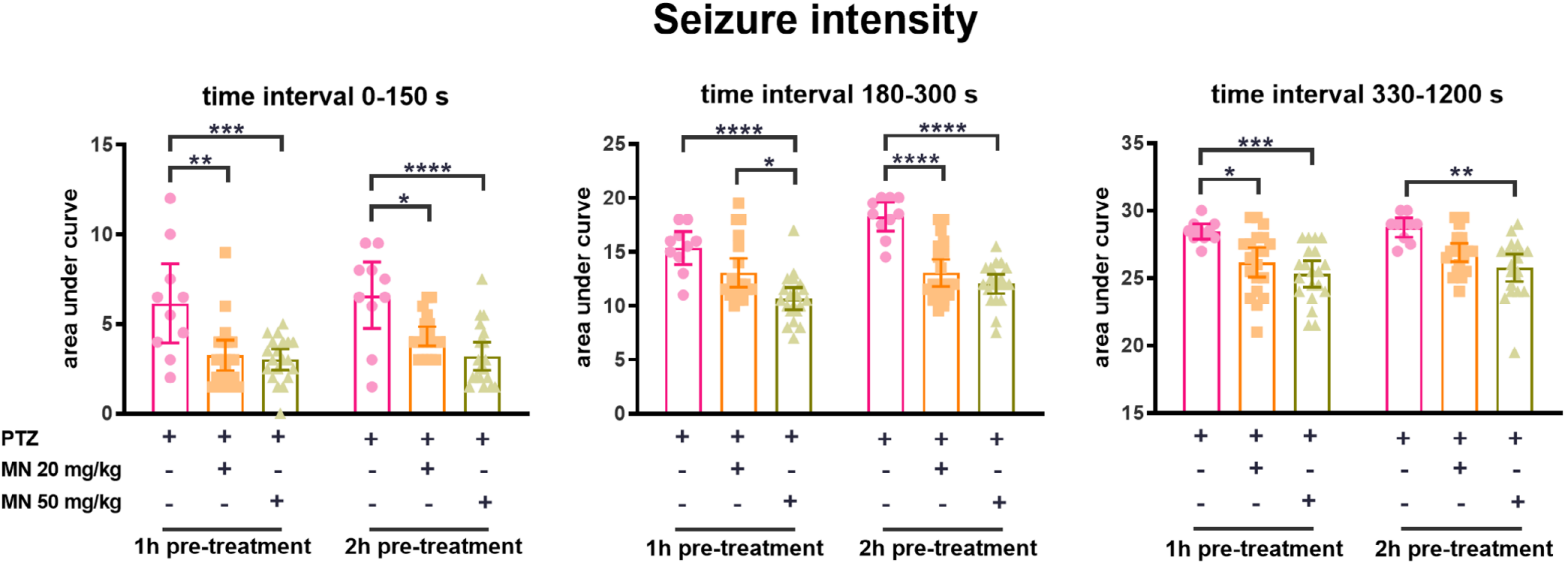
Effect of memantine on seizure severity. Seizure severity was evaluated as the total area under score curve (AUC) in three‐time intervals (0–150, 150–300, and 300–1200 s). Data were expressed as mean with 95% confidence interval (CI) and analyzed by two‐way ANOVA followed by Tukey's *post hoc* test. *p < 0.05, **p < 0.01, ***p < 0.001, and ****p < 0.0001.

Analysis of seizure progression revealed that vehicle‐treated animals experienced rapid seizure development within the first 300 s of PTZ exposure (Figure S3). In contrast, pre‐treatment with memantine at both doses markedly attenuated this progression, resulting in lower seizure scores throughout the observation period. Quantitative analysis confirmed that all memantine pre‐treatment regimens significantly reduced overall seizure severity across all tested time intervals compared to the vehicle/PTZ group (Figure 2) (time interval 0–150: *F*(2, 94) = 21.70, *p* < 0.0001; time interval 150–300: *F*(2, 94) = 35.59, *p* < 0.0001; time interval 300–1200: *F*(2, 94) = 17.91, *p* < 0.0001). Animals treated with vehicle/vehicle exhibited no seizure‐like behavior (data not shown).

### 3.2 Memantine Modulates PTZ‐Induced Alterations in NMDA Receptor Subunit Expression

**FIGURE 3:**
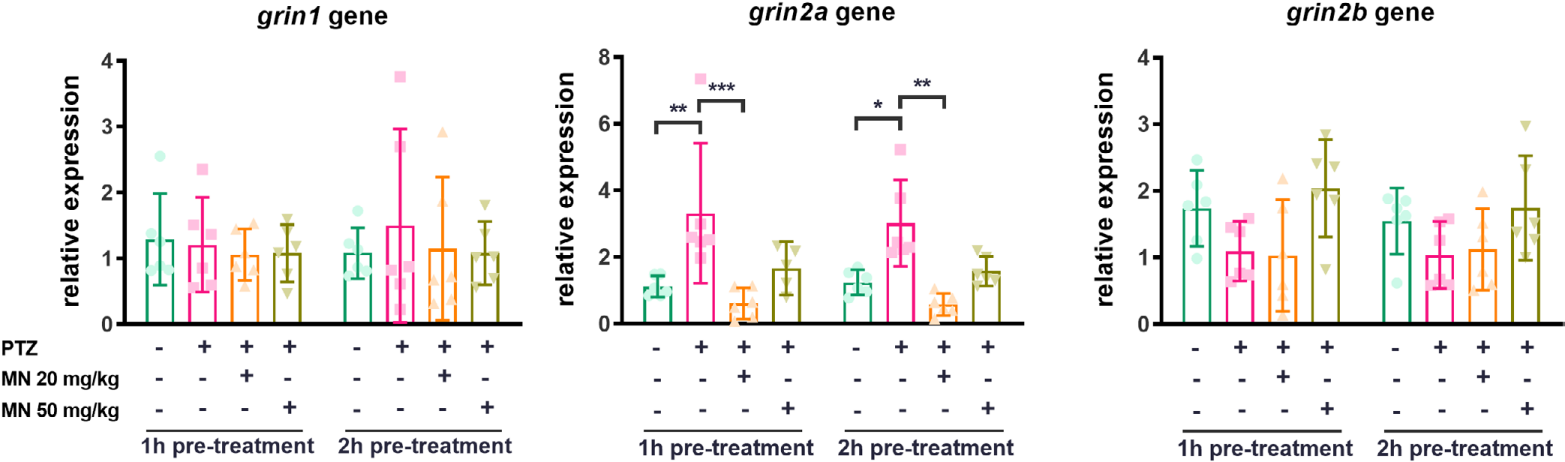
Expression of NMDA receptor subunits in the brain of zebrafish pre‐treated with memantine and submitted to PTZ‐induced seizure. Data were expressed as mean with 95% confidence interval (CI) and analyzed by two‐way ANOVA followed by Tukey's post hoc test. *p < 0.05; **p < 0.01, and ***p < 0.001. *N* = 6 samples/group, each sample comprising a pool of two zebrafish brains.

There was no difference among all groups regarding the expression of NMDA receptor subunits NR1 and NR2B (*grin1 and grin2b* genes, respectively) (Figure 3). However, a significant upregulation of the *grin2a* (NR2A) subunit was observed in the vehicle/PTZ group at both pre‐treatment timepoints (*F* (3, 39) = 17.23, *p* < 0.0001; Figure 3B). Pre‐treatment with MN (20 mg/kg) completely prevented this PTZ‐induced increase in *grin2a* expression at both 1 h. The higher dose of MN (50 mg/kg) produced only a partial, non‐significant reduction in *grin2a* upregulation.

### 3.3 Effects on Oxidative Stress and Antioxidant Defense

**FIGURE 4:**
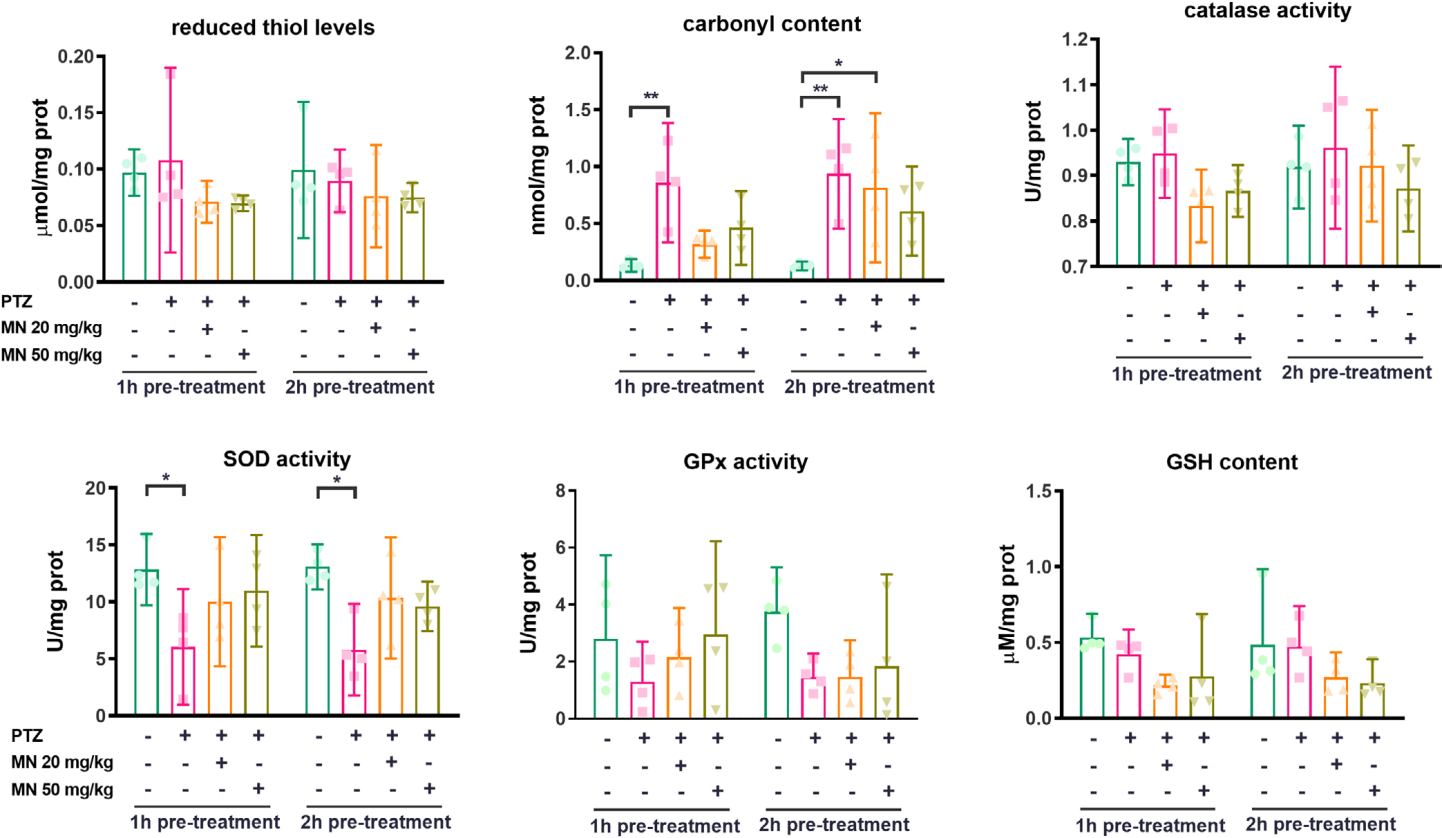
Oxidative stress and antioxidant defense in the brain of zebrafish pre‐treated with memantine and submitted to PTZ‐induced seizure. Data were expressed as mean ± with 95% confidence interval (CI) and analyzed by two‐way ANOVA followed by Tukey's post hoc test. *p < 0.05 and **p < 0.01. *N* = 4 samples/group, each sample comprising a pool of six zebrafish brains.

PTZ‐induced seizures significantly increased protein carbonylation (*F* (3, 24) = 13.14, *p* < 0.0001; Figure 4) and reduced SOD activity (*F*(3, 24) = 9.428, *p* = 0.0003) compared to the vehicle/vehicle control group. Pre‐treatment with MN showed a tendency to mitigate these changes, but the effects did not reach statistical significance. No significant differences were observed among groups in the levels of reduced thiols, catalase activity, GPx activity, or GSH content.

### 3.4 Effects on Anxiety‐Like Behavior

**FIGURE 5:**
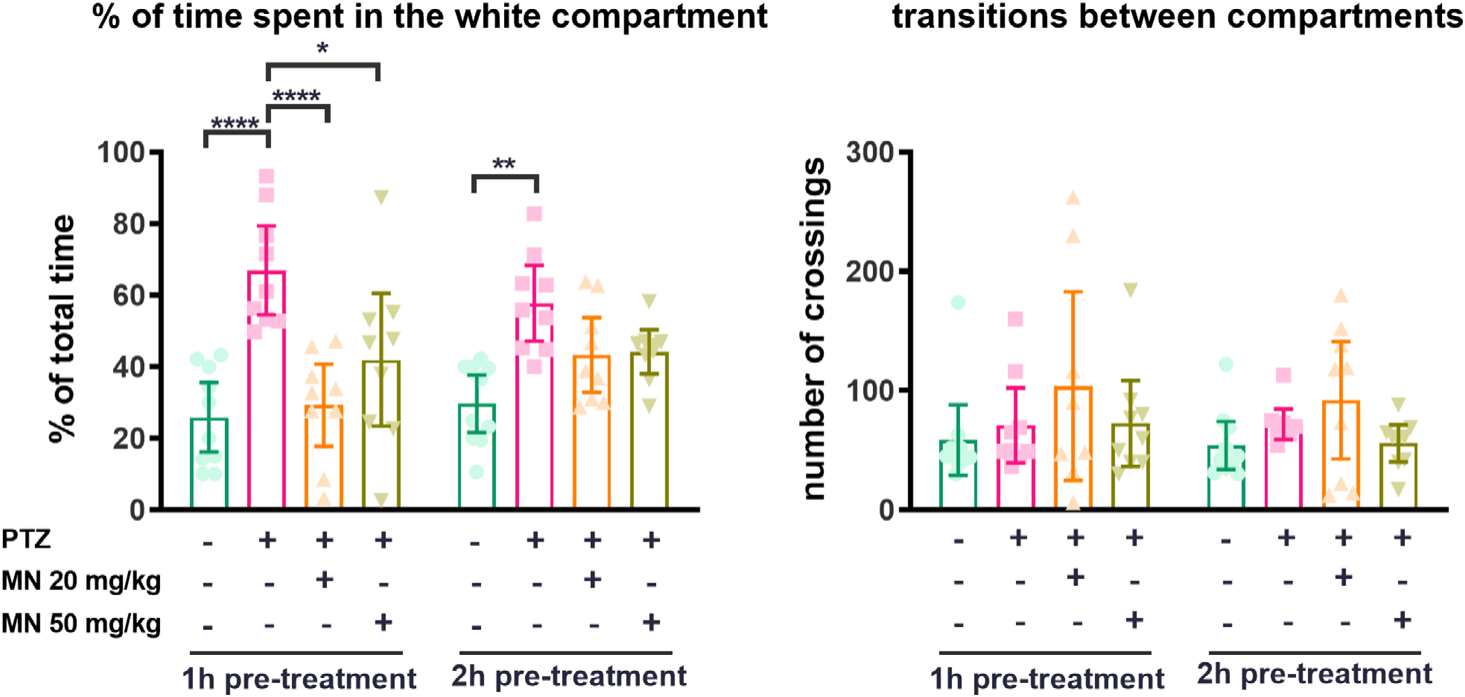
Effect of memantine on anxiety‐like behaviors induced by PTZ‐induced seizure. Data were expressed as mean ± SEM and analyzed by two‐way ANOVA followed by Tukey's post hoc test. *p < 0.05, **p < 0.01, and ****p < 0.0001. *N* = 10 animals/group.

In the light/dark test, PTZ exposure significantly increased anxiety‐like behavior, evidenced by an increased time spent in the white compartment in the vehicle/PTZ group at both pre‐treatment intervals (*F*(3, 66) = 17.98, *p* < 0.0001; Figure 5). Pre‐treatment with MN (both doses) 1 h before PTZ completely prevented this anxiogenic effect (20 mg/kg: *p* < 0.0001; 50 mg/kg: *p* = 0.0161 vs. respective PTZ group). This protective effect was not observed with the 2 h pre‐treatment regimen. The number of transitions between compartments was not altered by any treatment.

## 4 Discussion

The present study demonstrates a protective effect of memantine (MN) against PTZ‐induced seizures in adult zebrafish. Our principal findings indicate that MN pre‐treatment attenuates seizure severity, prevents PTZ‐induced upregulation of the GluN2A NMDA receptor subunit, mitigates oxidative stress markers, and normalizes anxiety‐like behaviors measured 24 h post‐seizure. The present observations posit that memantine confers protection in the PTZ‐induced seizure model not merely through acute NMDAR blockade, but by interrupting a cascade of linked pathological events: receptor dysregulation, excitotoxic stress, and consequent behavioral disruption. This multi‐target action underscores its potential as a repurposed therapeutic agent for epilepsy, capable of addressing both the ictal event and its neurobehavioral sequelae.

The anticonvulsant efficacy of memantine, evidenced by increased latency to tonic–clonic seizures and reduced overall seizure intensity, is consistent with its established pharmacology as an uncompetitive NMDAR blocker (Lipton 2006). However, its clinical translatability lies in its unique kinetic profile. Unlike high‐affinity blockers (e.g., ketamine or MK‐801), memantine's fast, voltage‐dependent action preferentially dampens pathological, tonic NMDAR activity—such as observed in epilepsy—while sparing physiological synaptic transmission (Parsons et al. 2008; Lipton 2006). Our data confirm that this selective antagonism is sufficient to suppress PTZ‐induced hyperexcitability, extending its proven efficacy from rodent models (Mareš and Mikulecká 2009; McLean et al. 1992) to zebrafish.

A pivotal finding is the specific, memantine‐sensitive upregulation of the *grin2a* (GluN2A) subunit. This is not a mere correlate of seizure activity but may represent a feedforward mechanism promoting hyperexcitability. Increased GluN2A incorporation alters NMDAR kinetics, favoring increased calcium permeability and excitatory postsynaptic currents—properties that can lower the seizure threshold and contribute to epileptogenesis (Chen et al. 2022; Ambrogini et al. 2019). By preventing this maladaptive plasticity, memantine may act prophylactically, disrupting a key molecular step that links an acute seizure to lasting network hyperexcitability and epileptogenesis. This moves its role beyond symptomatic control toward potential disease modification.

The observed oxidative damage, such as increased protein carbonylation and reduced SOD activity, is a likely downstream consequence of NMDAR‐driven calcium influx, which activates pro‐degenerative pathways involving nNOS, calpain I, and mitochondrial dysfunction (Chen et al. 2022; Ambrogini et al. 2019). Calpain I activation may increase lysosomal membrane permeability (LMP), promoting ROS formation and oxidative stress with subsequently lysosomal cell death (Chen et al. 2022). While memantine pre‐treatment showed a non‐significant trend in mitigating these markers, this tendency is mechanistically coherent: by reducing the primary excitotoxic drive, memantine indirectly alleviates the resulting oxidative burden. Therefore, its primary antioxidant effect may be upstream, via receptor blockade, rather than through direct scavenging activity.

Its well‐documented in literature that epilepsy is associated with several neuropsychiatry and cognitive disorders (Minjarez et al. 2017; Tolchin et al. 2020; Operto et al. 2023). Psychiatric comorbidities are threefold more frequent in patients with epilepsy than in the normal population (Tellez‐Zenteno et al. 2007). Therefore, the post‐seizure behavioral phenotype observed here—increased time spent in the aversive white zone—likely reflects a disruption of MNDA‐dependent neural circuits governing threat assessment, not merely anxiety. This is in alignment with observations in rodent models where seizure activity can impair fear processing (Detour et al. 2005). Moreover, in the pilocarpine‐induced seizure model, epileptic rats stayed longer periods in the open arms of the elevated‐plus maze when compared to control ones (Detour et al. 2005; De Oliveira et al. 2008). That memantine prevented this dysfunction suggests it preserves the integrity of these circuits, possibly by limiting seizure‐associated excitotoxicity in key limbic regions. This highlights a benefit beyond motor seizure suppression: the protection of cognitive‐affective networks vulnerable to post‐ictal damage.

In conclusion, our findings frame memantine's action in the PTZ model as a coordinated intervention at multiple levels of seizure pathology: (1) *acute channel blockade* to reduce hyperexcitability, (2) *prevention of maladaptive NMDAR subunit plasticity* that could promote epileptogenesis, and (3) *preservation of neural circuits* underlying affective behavior. This multi‐target profile, combined with its established clinical safety and tolerability, provides a strong rationale for investigating memantine as an adjunctive therapy in epilepsy. Future studies should focus on its effects in chronic models to evaluate its true disease‐modifying potential and its efficacy against comorbid cognitive and affective impairments.

## Author Contributions

**Kamila Cagliari Zenki:** conceptualization, methodology, data curation, investigation, writing – original draft, formal analysis. **Eduardo Kalinine:** investigation, methodology. **Ben Hur Marins Mussulini:** investigation, methodology, writing – original draft. **Thainá Garbino dos Santos:** investigation, methodology, writing – original draft. **Lucia von Mengden:** investigation, methodology. **Fábio Klamt:** writing – review and editing. **Suelen Baggio:** investigation, methodology, writing – original draft. **Ana Carolina de Moura:** investigation, methodology. **Ana Beatriz Gorini da Veiga:** methodology, writing – original draft. **Diogo Losch de Oliveira:** conceptualization, methodology, data curation, formal analysis, writing – original draft, writing – review and editing, funding acquisition, project administration.

## Conflicts of Interest

The authors declare no conflicts of interest.

## Supporting information


**Figure S1:** Acute effects of memantine on locomotor activity in naïve zebrafish.
**Figure S2:** Experimental design for evaluating the anticonvulsant and neuroprotective effects of memantine.
**Figure S3:** Memantine attenuates the progression of PTZ‐induced seizures.
**Table S1:** Detailed statistical reports for all two‐way ANOVA and normality D'Agostino‐Pearson tests.

## Data Availability

The data that support all findings of this study are available from the corresponding author upon reasonable request.
