# Supplementary material for: Memantine Confers Multi‐Target Protection in a Zebrafish Seizure Model: Attenuating Epileptic Behavior, GluN2A Overexpression, and Oxidative Stress
Source: J Neurochem. 2026 Jan 2;170(1):e70345. doi: 10.1111/jnc.70345 (PMC12758096; doi:10.1111/jnc.70345)
Supplement: Supplementary file 1 — Figure S1: Acute effects of memantine on locomotor activity in naïve zebrafish. Figure S2: Experimental design for evaluating the anticonvulsant and neuroprotective effects of memantine. Figure S3: Memantine attenuates the progression of PTZ‐induced seizures. Table S1: Detailed statistical reports for all two‐way ANOVA and normality D'Agostino‐Pearson tests. [file JNC-170-0-s001.pdf]

Journal of Neurochemistry

Supplementary material

Memantine confers multi-target protection in a zebrafish seizure model: attenuating epileptic behavior, GluN2A overexpression, and oxidative stress.

Kamila Cagliari Zenki[a,b], Eduardo Kalinine[b], Ben Hur Marins Mussulini[a,b,e], Thainá Garbino dos Santos[a,b], Lucia von Mengden[b,c], Fábio Klamt[b,c], Suelen Baggio[a,b,e], Ana Carolina de Moura[d], Ana Beatriz Gorini da Veiga[d], Diogo Losch de Oliveira* [a,b]

[a] Laboratory of Neural Development, Department of Biochemistry, Instituto de Ciências Básicas da Saúde, Universidade Federal do Rio Grande do Sul, Porto Alegre, Brazil.
[b] Programa de Pós-graduação em Ciências Biológicas: Bioquímica, Instituto de Ciências Básicas da Saúde, Universidade Federal do Rio Grande do Sul, Brazil.
[c] Laboratory of Cellular Biochemistry, Department of Biochemistry, Instituto de Ciências Básicas da Saúde, Universidade Federal do Rio Grande do Sul, Brazil.
[d] Laboratory of Molecular Biology, Universidade Federal de Ciências da Saúde de Porto Alegre – UFCSPA, Brazil.
[e] Centre of New Technologies, University of Warsaw, Warszawa, Poland

* Address for correspondence:
Diogo Losch de Oliveira
Department of Biochemistry - ICBS
Universidade Federal do Rio Grande do Sul
Rua Ramiro Barcelos 2600 - Anexo
Zip code: 90035-003
Porto Alegre - RS - Brazil
Phone: +55 51 33085555
Fax: +55 51 33085540
ORCID 0000-0002-9028-6959
E-mail: losch@ufrgs.br

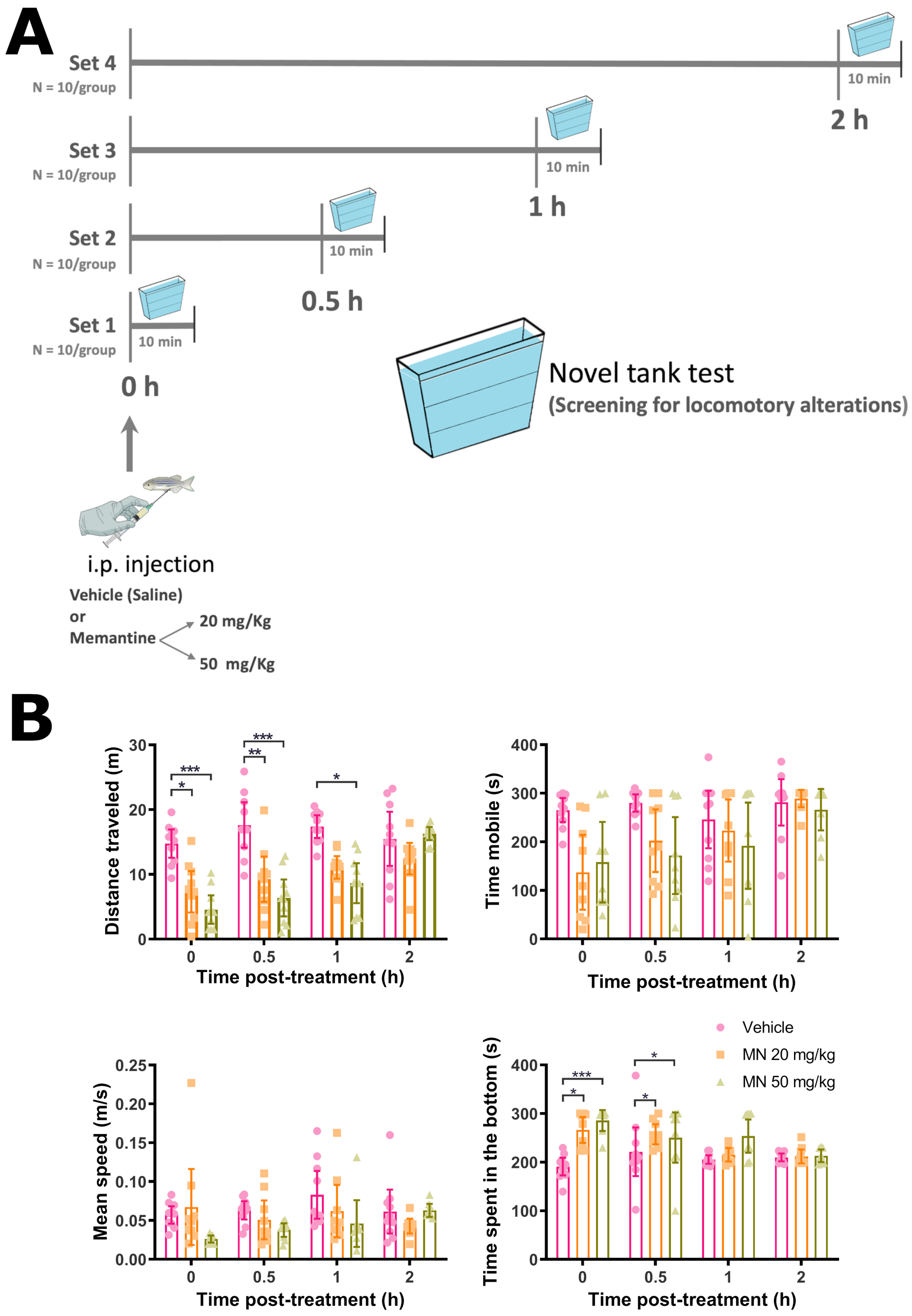

Supplementary Figure 1. Acute effects of memantine on locomotor activity in naïve zebrafish. (A) Schematic timeline of the experimental design for acute behavioral assessment. **(B)** Locomotor and exploratory profiles of zebrafish following intraperitoneal injection of vehicle or memantine (20 or 50 mg/kg). Behavior was quantified in the novel tank test at the following time points 0, 0.5, 1, or 2 h after memantine injection over a 10-minute session. Data are presented as mean with 95% confidence interval (CI; n=10 fish/group).

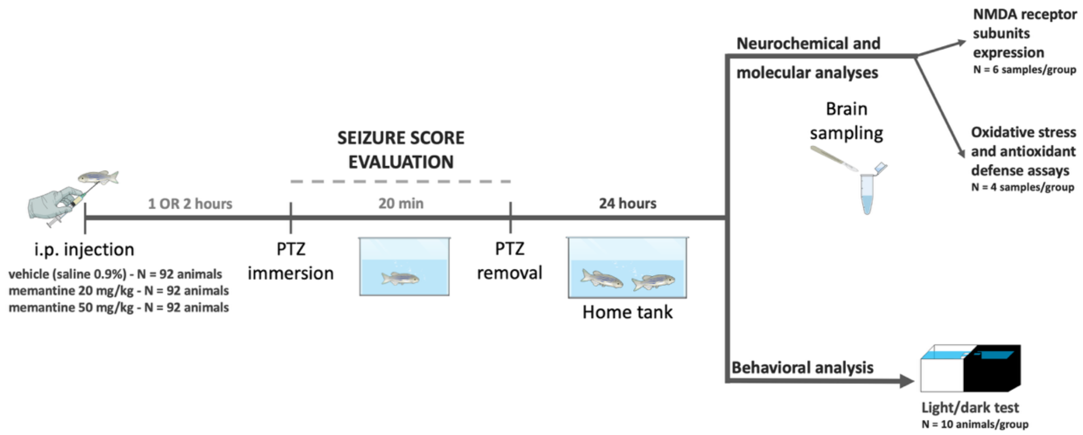

Supplementary Figure 2. Experimental design for evaluating the anticonvulsant and neuroprotective effects of memantine. Schematic overview of the treatment protocol. Adult zebrafish were pre-treated with vehicle or memantine (20 or 50 mg/kg, i.p.) 1 or 2 hours prior to induction of seizures with 10 mM pentylenetetrazole (PTZ). Seizure behavior was scored live for 20 minutes according to established criteria (Mussulini et al., 2013). Twenty-four hours post-ictus, animals were allocated into separate cohorts for neurochemical/molecular analyses and behavioral phenotyping.

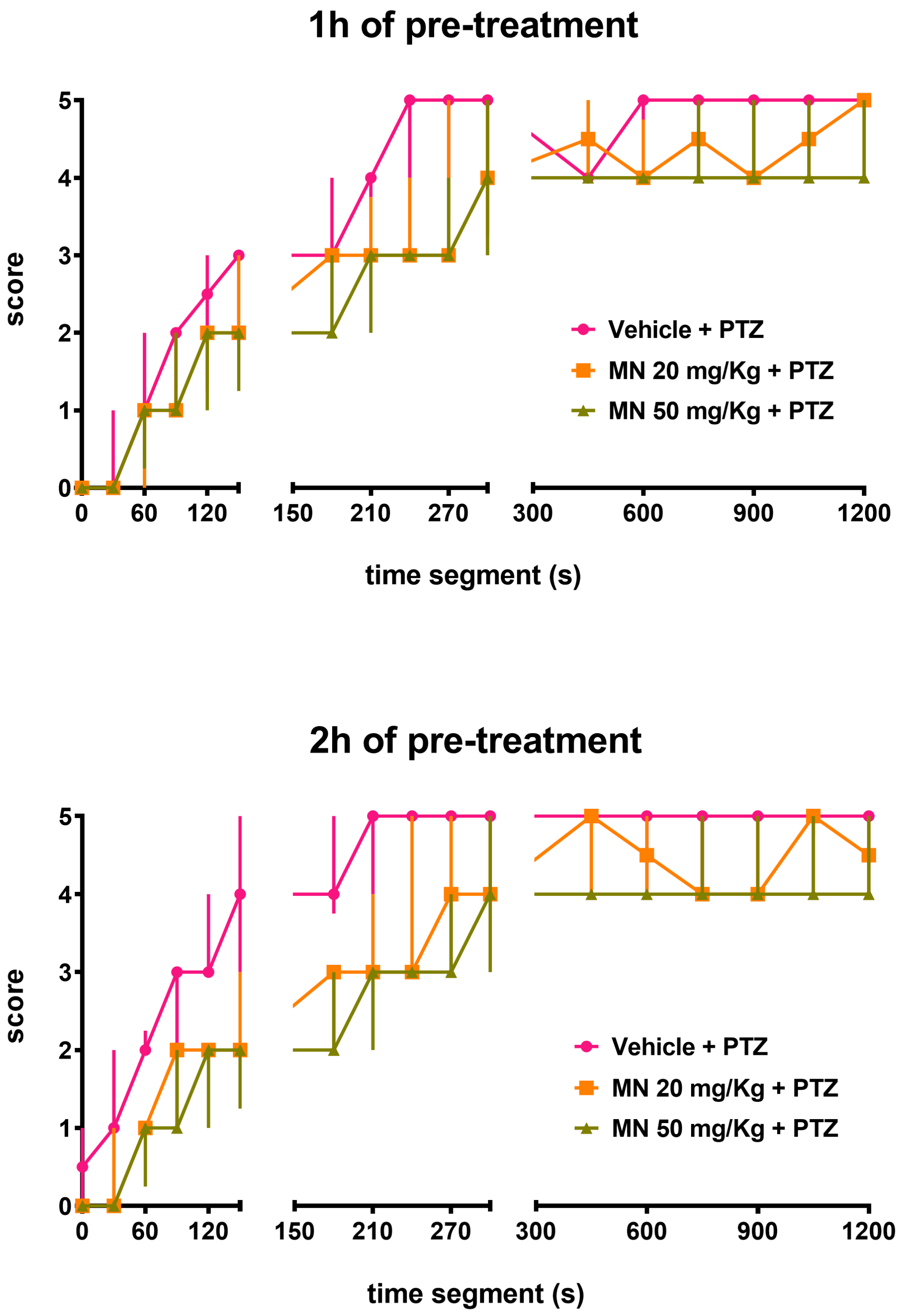

Supplementary Figure 3. Memantine attenuates the progression of PTZ-induced seizures. Temporal profile of seizure scores in zebrafish pre-treated with memantine or vehicle before PTZ exposure. Data are presented as median with interquartile range. For quantitative analysis, the area under the curve (AUC) for seizure severity was calculated across three defined time intervals: 0-150 s, 150-300 s, and 300-1200 s.

Sample size calculation and randomization method

The sample size was determined a priori using the "pwrss" package (v1.0.0) in R, based on the following criteria: number of factor A = 3, number of factor B = 2, α = 0.05, test power = 0.8, effect size = 0.5. Based on this calculation, animals were randomly assigned to experimental groups using a computer-generated sequence created with the randomizr (v1.0.0) and dplyr (v1.1.4) packages. The complete R script for both procedures is provided below.

```
# Installing and loading packages
install.packages("randomizr")
library(randomizr)
install.packages("dplyr")
library(dplyr)
install.packages("pwrss")
library(pwrss)

###############################################
# Pilot experiment for sedative effects of MN #
###############################################

# Assigning fish for each treatment group
N <- 120 # total sample size
pilot_group_assignment <- complete_ra(
  N,
  num_arms = 4,
  conditions = c("0_hour", "0.5_hour", "1_hour", "2_hour")
  )

# Creating the randomization table
pilot_randomization_table <- data.frame(
  fish = paste("Fish",120),
  treatment_group = pilot_group_assignment
)

# Verifying the number of fish assigned for each group
table(pilot_randomization_table$treatment_group)

############################################################
# Sample size calculation for anticonvulsive effects of MN #
############################################################

# Estimate sample size using ANOVA
n_total <- power.f.ancova(eta.squared = 0.026,
                          factor.levels = c(3,2),
                          alpha = 0.05,
                          power = .80)
# This estimation resulted in a sample size of 366 animals, which resulted in 91.5 animals/group. This number was rounded to 92 animals/group = totalizing 368 animals.

################################################
# Experiments for anticonvulsive effects of MN #
################################################

# Assigning fish for each treatment group
N <- n_total[["n.total"]]+2 # total sample size calculated by 'pwrss' + 2 animals (for rounding)
anticonvulsive_group_assignment <- complete_ra(
```

```
  N,
  num_arms = 8,
  conditions                                                           =
c("VEH_1h","VEH_2h","PTZ_1h","PTZ_2h","MN20_1h","MN20_2h","MN50_1h","M
N50_2h"))

# Creating the randomization table
anticonvulsive_randomization_table <- data.frame(
  fish = paste("Fish",1:368),
  treatment_group = anticonvulsive_group_assignment
)

# Verifying the number of fish assigned for each group
table(anticonvulsive_randomization_table$treatment_group)

# Defining assays
assays <- c("behavior","oxidative_stress","PCR")

# Assigning treatment-assigned-fish for each assay
anticonvulsive_randomization_table$assigned_assay <- block_ra(
  blocks = anticonvulsive_randomization_table$treatment_group,
  conditions = assays,
  prob_each = c(20/92, 48/92, 24/92)
)

# Verifying the number fish from each treatment group assigned for each
assay
sample_size_table <- anticonvulsive_randomization_table %>%
  group_by(treatment_group) %>%
  count(assigned_assay)
sum(sample_size_table$n)
```

Detailed statistical reports

Supplementary table 1. Detailed statistical reports for all two-way ANOVA and normality D'Agostino-Pearson tests.

PTZ-induced seizures

Groups

Latency to score 4

**ANOVA table**	**F (DFn, DFd)**	**P value**	**D'Agostino-Pearson test**	**PTZ**	**MN20**	**MN50**
Interaction	F (2, 94) = 0,3718	P=0,6905	K2	1,665	4,357	5,104
Row Factor	F (1, 94) = 4,240	P=0,0423	P value	0,4349	0,1132	0,0721
Column Factor	F (2, 94) = 13,46	P<0,0001	Passed normality test (alpha=0.05)?	Yes	Yes	Yes

Latency to return to score 0

**ANOVA table**	**F (DFn, DFd)**	**P value**	**D'Agostino-Pearson test**	**PTZ**	**MN20**	**MN50**
Interaction	F (2, 62) = 1,536	P=0,2233	K2	3,198	5,963	0,8320
Row Factor	F (1, 62) = 1,817	P=0,1826	P value	0,2021	0,0507	0,6597
Column Factor	F (2, 62) = 8,667	P=0,0005	Passed normality test (alpha=0.05)?	Yes	Yes	Yes

Seizure intensity – time interval 0-150

**ANOVA table**	**F (DFn, DFd)**	**P value**	**D'Agostino-Pearson test**	**PTZ**	**MN20**	**MN50**
Interaction	F (2, 94) = 0,6201	P=0,5401	K2	0,08063	3,595	4,283
Row Factor	F (1, 94) = 2,148	P=0,1461	P value	0,9605	0,2682	0,1175
Column Factor	F (2, 94) = 21,70	P<0,0001	Passed normality test (alpha=0.05)?	Yes	Yes	Yes

Seizure intensity – time interval 150-300

**ANOVA table**	**F (DFn, DFd)**	**P value**	**D'Agostino-Pearson test**	**PTZ**	**MN20**	**MN50**
Interaction	F (2, 94) = 2,596	P=0,0799	K2	1,543	5,713	0,6224
Row Factor	F (1, 94) = 8,109	P=0,0054	P value	0,4623	0,0575	0,7326
Column Factor	F (2, 94) = 35,59	P<0,0001	Passed normality test (alpha=0.05)?	Yes	Yes	Yes

Seizure intensity – time interval 300-1200

**ANOVA table**	**F (DFn, DFd)**	**P value**	**D'Agostino-Pearson test**	**PTZ**	**MN20**	**MN50**
Interaction	F (2, 94) = 0,09630	P=0,9083	K2	0,1259	3,195	4,601
Row Factor	F (1, 94) = 1,611	P=0,2075	P value	0,9390	0,2024	0,1002
Column Factor	F (2, 94) = 17,91	P<0,0001	Passed normality test (alpha=0.05)?	Yes	Yes	Yes

NMDA receptor subunit expression

Groups

grin1

ANOVA table	F (DFn, DFd)	P value	D'Agostino-Pearson test	Veh	PTZ	MN20	MN50
Interaction	F (3, 40) = 0,2218	P=0,8807	K2	4,4284	4,941	4,468	0,3876
Row Factor	F (1, 40) = 0,04161	P=0,8394	P value	0,0971	0,0845	0,0939	0,8238
Column Factor	F (3, 40) = 0,3201	P=0,8108	Passed normality test (alpha=0.05)?	Yes	Yes	Yes	Yes

grin2a

ANOVA table	F (DFn, DFd)	P value	D'Agostino-Pearson test	Veh	PTZ	MN20	MN50
Interaction	F (3, 39) = 0,1070	P=0,9555	K2	3,416	3,982	1,200	1,457
Row Factor	F (1, 39) = 0,07268	P=0,7889	P value	0,1812	0,1269	0,5489	0,4826
Column Factor	F (3, 39) = 17,23	P<0,0001	Passed normality test (alpha=0.05)?	Yes	Yes	Yes	Yes

grin2b

ANOVA table	F (DFn, DFd)	P value	D'Agostino-Pearson test	Veh	PTZ	MN20	MN50
Interaction	F (3, 40) = 0,2293	P=0,8755	K2	1,803	2,782	1,618	1,194
Row Factor	F (1, 40) = 0,4179	P=0,5217	P value	0,4060	0,2275	0,4453	0,5504
Column Factor	F (3, 40) = 5,610	P=0,0026	Passed normality test (alpha=0.05)?	Yes	Yes	Yes	Yes

Oxidative stress and antioxidant defense

Reduced thiol levels

ANOVA table	F (DFn, DFd)	P value	D'Agostino-Pearson test	Veh	PTZ	MN20	MN50
Interaction	F (3, 24) = 0,3682	P=0,7766	K2	3,424	3,821	4,003	3,146
Row Factor	F (1, 24) = 0,02596	P=0,8734	P value	0,148	0,1102	0,0697	0,2074
Column Factor	F (3, 24) = 2,483	P=0,0851	Passed normality test (alpha=0.05)?	Yes	Yes	Yes	Yes

Carbonyl content

ANOVA table	F (DFn, DFd)	P value	D'Agostino-Pearson test	Veh	PTZ	MN20	MN50
Interaction	F (3, 24) = 1,610	P=0,2131	K2	3,373	1,232	2,905	2,424
Row Factor	F (1, 24) = 4,249	P=0,0503	P value	0,1852	0,5401	0,2339	0,2976
Column Factor	F (3, 24) = 13,14	P<0,0001	Passed normality test (alpha=0.05)?	Yes	Yes	Yes	Yes

Catalase activity

ANOVA table	F (DFn, DFd)	P value	D'Agostino-Pearson test	Veh	PTZ	MN20	MN50
Interaction	F (3, 24) = 0,9253	P=0,4436	K2	0,6835	2,742	0,8210	1,432
Row Factor	F (1, 24) = 1,094	P=0,3059	P value	0,7105	0,2539	0,6633	0,4886
Column Factor	F (3, 24) = 3,073	P=0,0469	Passed normality test (alpha=0.05)?	Yes	Yes	Yes	Yes

SOD activity

ANOVA table	F (DFn, DFd)	P value	D'Agostino-Pearson test	Veh	PTZ	MN20	MN50
Interaction	F (3, 24) = 0,1719	P=0,9144	K2	2,052	0,3428	0,6767	0,7480
Row Factor	F (1, 24) = 0,07610	P=0,7850	P value	0,3584	0,8425	0,7129	0,6880
Column Factor	F (3, 24) = 9,428	P=0,0003	Passed normality test (alpha=0.05)?	Yes	Yes	Yes	Yes

GPx activity

ANOVA table	F (DFn, DFd)	P value	D'Agostino-Pearson test	Veh	PTZ	MN20	MN50
Interaction	F (3, 24) = 0,8692	P=0,4707	K2	1,419	0,6865	0,1817	3,368
Row Factor	F (1, 24) = 0,1228	P=0,7291	P value	0,4919	0,7094	0,9132	0,1857
Column Factor	F (3, 24) = 2,772	P=0,0634	Passed normality test (alpha=0.05)?	Yes	Yes	Yes	Yes

GSH content

ANOVA table	F (DFn, DFd)	P value	D'Agostino-Pearson test	Veh	PTZ	MN20	MN50
Interaction	F (3, 24) = 0,2238	P=0,8789	K2	4,768	0,3381	2,095	3,340
Row Factor	F (1, 24) = 0,002871	P=0,9577	P value	0,0922	0,8445	0,3508	0,2364
Column Factor	F (3, 24) = 4,946	P=0,0082	Passed normality test (alpha=0.05)?	Yes	Yes	Yes	Yes

Anxiety-like behavior

Time spent in the white compartment

ANOVA table	F (DFn, DFd)	P value	D'Agostino-Pearson test	Veh	PTZ	MN20	MN50
Interaction	F (3, 66) = 1,806	P=0,1546	K2	3,264	1,493	0,7492	3,913
Row Factor	F (1, 66) = 0,5963	P=0,4428	P value	0,2217	0,4739	0,6876	0,1414
Column Factor	F (3, 66) = 17,98	P<0,0001	Passed normality test (alpha=0.05)?	Yes	Yes	Yes	Yes

Transitions between compartments

ANOVA table	F (DFn, DFd)	P value	D'Agostino-Pearson test	Veh	PTZ	MN20	MN50
Interaction	F (3, 65) = 0,1176	P=0,9495	K2	2,85	2,46	1,750	2,75
Row Factor	F (1, 65) = 0,4955	P=0,4840	P value	0,2127	0,3771	0,4168	0,3159
Column Factor	F (3, 65) = 2,456	P=0,0708	Passed normality test (alpha=0.05)?	Yes	Yes	Yes	Yes

Veh group: Animals treated with vehicle (NaCl 0.9% solution).
PTZ group: Animals treated submitted to PTZ-induced seizure.
MN20 group: Animals treated with memantine 20 mg/kg and submitted to PTZ-induced seizure.
MN50 group: Animals treated with memantine 50 mg/kg and submitted to PTZ-induced seizure.
